# Laparoscopic Extravesical Ureteral Reimplantation: Technique

**DOI:** 10.1155/2008/567980

**Published:** 2008-08-18

**Authors:** John-Paul Capolicchio

**Affiliations:** Division of Urology, The Montreal Children's Hospital, McGill University Health Centre, 2300 Tupper, Suite C527, Montreal, QC, Canada H3H 1P3; Shriners Hospital for Children, 1529 Cedar Avenue, Montreal, QC, Canada H3G1A6

## Abstract

Laparoscopic extravesical ureteral reimplantation in children is currently a technically demanding procedure with sparse literature to aid in mastering the learning curve. We present our most recent technique and lessons learned after 20 cases in children 4–15 years of age. The literature is also reviewed to encapsulate the current state-of-the-art.

## 1. INTRODUCTION

Open extravesical ureteral reimplantation is a successful and well-tolerated procedure with a proven track record in the surgical management of vesicoureteric reflux (VUR). Nevertheless, the relentless pursuit of minimally invasive ideals has led to the development of alternatives. Most recently, the endoscopic injection techniques have become quite popular, but concerns remain over the success rate and long-term efficacy. Thus, the laparoscopic approach offers another option which improves on the open procedure with better cosmesis and convalescence, while providing a durable and successful procedure compared to injection therapy.

Despite multiple reports in the early 1990s of experimental surgery in animal models [1–3] and few cases in humans [4, 5], it was not until the seminal contributions of the late Leo Fung in 2000 that the technical aspects and outcomes of the procedure were documented [6]. Since then, the peer reviewed literature has been sparse, such that the learning curve of this procedure is not well established. We review our current experience and lessons learned in the process.

## 2. MATERIALS AND METHODS

### 2.1. Patients

A total of 20 children aged 4–15 years (mean 7.3 years) have undergone laparoscopic extravesical ureteral reimplantation over a 5-year period. The subjects were mostly female (15 of 20) with 11 (55%) cases being bilateral. All cases were diagnosed with VUR after urinary infection and the indication for surgery included breakthrough infection in 18 of 20 and persistent high-grade VUR in 2 of 20.

The highest grade of reflux per patient ranged from 2 to 4, with only 1 case of unilateral grade 2, that being a case of failed injection therapy. The distribution of VUR per patient by highest grade was grade 4 in 7 patients (35%), grade 3 in 10 (50%), and grade 2 in 3 (15%). Megaureters, duplicated ureters, and neurogenic bladders were excluded initially. Previous open ureteral surgery remains an exclusion criterion. Bilateral cases are selected such that one side is not high-grade VUR, so as to minimize the risk of urinary retention. This hypothesis is based on the postulates that bladder dysfunction should not occur with unilateral extravesical dissection, and that high-grade VUR is a risk factor for postoperative bladder dysfunction [7].

The postoperative follow-up regimen includes a routine abdomino-pelvic ultrasound 1 month after surgery and a voiding cystourethrogram 3 months after surgery, with maintenance of antibiotic prophylaxis until the VCUG is done. In the absence of new findings on the first post-op ultrasound, another routine abdomino-pelvic ultrasound is planned 1 year after surgery.

### 2.2. Technique

We find it useful for learning purposes to divide the case into four specific tasks: 1-access, 2-uretero-vesical junction exposure, 3-detrusor tunnel dissection, and 4-tunnel suturing.

### 2.3. Access

**Figure 1:**
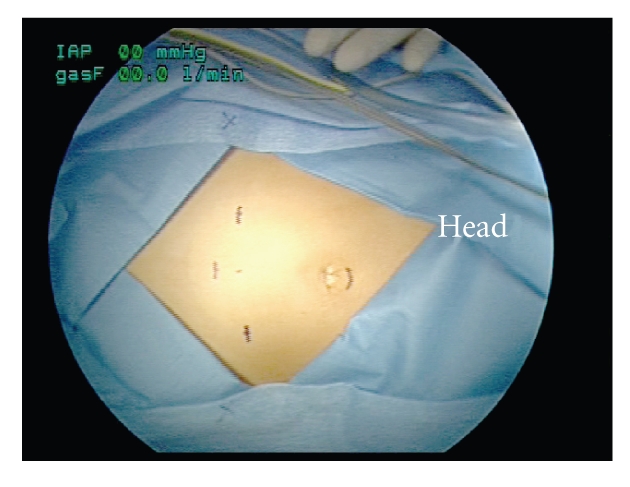
Port placement.

A 4-port approach is utilized with the patient in Trendelenburg position, legs spread apart, and the arms tucked in at the side. A sterile Foley catheter is placed in the operative field and controlled with a Toomey syringe. A mechanical bowel preparation can be helpful in patients with constipation. The first port is supraumbilical, and the 3 others form an arc along the level of the anterior-superior iliac spine (Figure 1). The level of this arc is adjusted downwards as the patient age increases and the bladder is further from the umbilicus. The arc is formed by 1 port on either lateral edge of the rectus and 1 midline port. All ports are 5 mm except for the 3 mm inferior midline port. A zero degree telescope is placed at the upper edge of the umbilicus with 2 video monitors at the foot of the bed. The surgeon and assistant are contralateral to the ureter with the assistant holding the camera while seated caudal to the surgeon.

### 2.4. Exposure of the ureter

**Figure 2:**
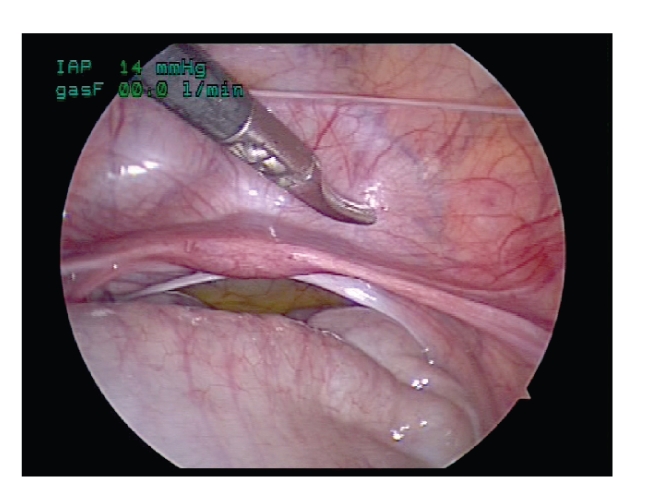
The peritoneal envelope is opened just adjacent to the bladder.

**Figure 3:**
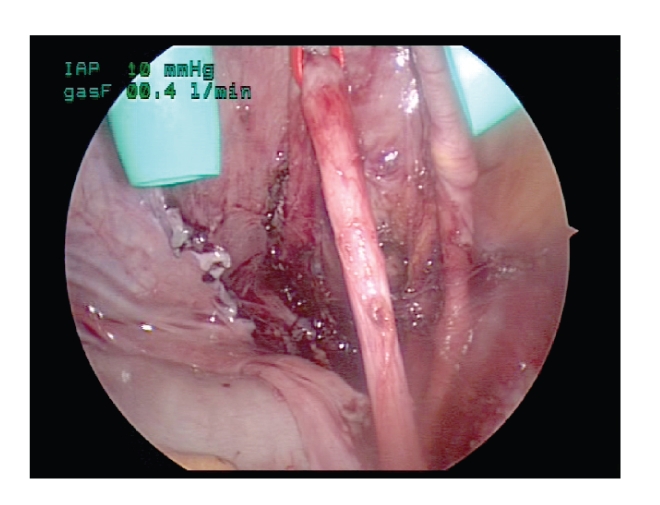
The ureter is mobilized while the assistant maintains traction with a vessel loop.

The peritoneal envelope is opened just adjacent to the bladder, caudal to the Fallopian tube or vas deferens in the male (Figure 2). The round ligament is also divided to further open the peritoneal window. The ureter is readily identified by *blunt* dissection adjacent to the bladder, often with the superior vesical artery coursing parallel. The assistant then controls the ureter with a vessel loop (Figure 3) through the inferior midline port which provides the exposure needed for the surgeon to mobilize the ureter from the pelvic brim to the uretero-vesical junction.

### 2.5. Detrusor tunnel dissection

**Figure 4:**
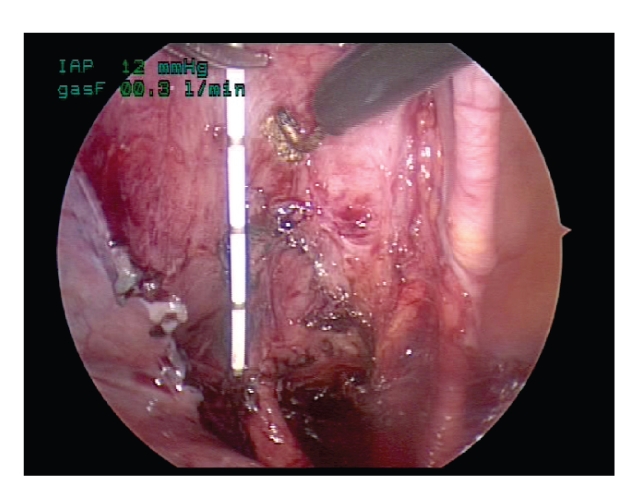
The tunnel should be oriented vertically and its length can be measured with a piece of ureteral catheter acting as a ruler.

The bladder is partially filled via the Toomey syringe, and the planned detrusor tunnel is exposed with 2 percutaneously passed suspension sutures of 3–0 silk. A fascial closure device is utilized to pass the hitch stitch percutaneously after an appropriate exit site has been chosen. These hitch stitches are placed on either side of the apex of the planned tunnel and should be angled so as to provide a distraction force to the edges of the detrusor tunnel. The direction of the planned tunnel should be oriented vertically and its length can be measured with a piece of ureteral catheter acting as a ruler (Figure 4). The direction of the tunnel is crucial in determining subsequent ergonomics of both tunnel dissection and suturing.

**Figure 5:**
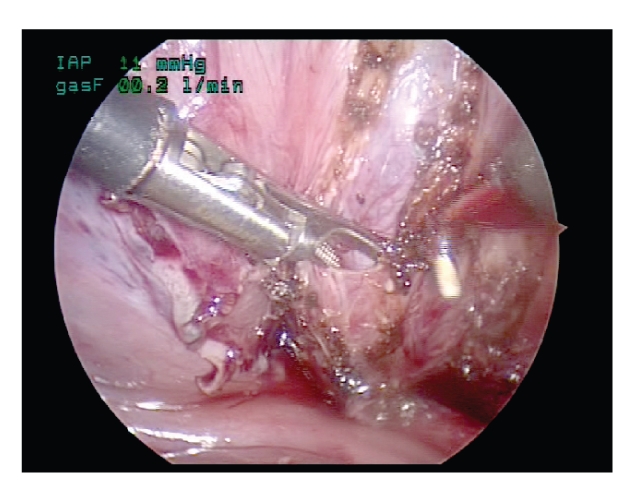
Dissection of the submucosal tunnel.

The planned tunnel is scored with cautery and the superficial detrusor then cauterized. The remaining detrusor fibers are sharply divided with scissors from apex of the tunnel towards the ureterovesical junction (Figure 5). Careful hemostasis is needed to maintain exposure. The dissection on the right side is easier for a right-handed surgeon. The left side tunnel dissection is done with the scissor in the left lateral port and controlled with the left hand. The right angle forcep and right angle electrocautery can also be very helpful during the dissection around the ureterovesical junction. The amount of mucosal bulging can be adjusted by the volume of bladder filling or via the intraperitoneal insufflation pressure. Any holes in the mucosa can be closed with a figure of eight stitch of 5–0 plain. The mucosal edges of the detrusor tunnel are not undermined.

### 2.6. Tunnel suturing

**Figure 6:**
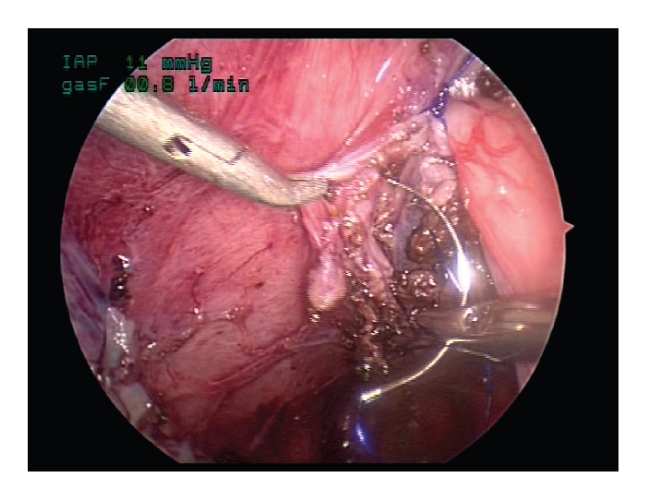
Suturing is back-hand with the instruments medial to the ureter.

The ureter is then advanced into the detrusor trough, and the first stitch defines the neohiatus. That stitch is then held by the assistant, while the remainder of the detrusor tunnel is closed. The bladder is emptied, and the detrusor tunnel closed with interrupted 5–0 PDS suture on a RB1 needle. The suture is controlled with a 3 mm angled forcep and 3 mm needle driver. All suturing is back-hand with the instruments medial to the ureter (Figure 6). Interrupted stitches alternate from each end of the tunnel with the last stitch placed in the mid-tunnel so as to avoid inadvertent suture of the underlying ureter. Having completed the reimplantation, the bladder traction sutures are released, and the bladder is cycled to confirm the absence of a urine leak or kinking of the ureter at the neohiatus. A closed suction drain is left in cases, where the mucosa was opened. The bladder catheter is removed the following morning.

## 3. RESULTS

All patients who have been studied postoperatively with a voiding cystourethrogram (VCUG) have had resolution of reflux, with 2 cases refusing the post-op VCUG and 1 being lost to follow-up. One case developed de novo contralateral grade 2 VUR. Three cases were converted to open surgery, the first 2 cases, both bilateral, because surgical time had surpassed 4 hours. Case 7 had a nonneurogenic neurogenic bladder with a severely hypertrophied detrusor which made tunnel dissection difficult.

The first 5 cases, 4 of which were bilateral, can be considered the learning curve with operative times falling consistently below 3 hours for a unilateral case, and 5 hours for a bilateral case thereafter. Mucosal perforation remains the main determinant of operative time, in its absence the operative time averages 2 hours per ureter. Mucosal perforation also remains the main determinant of hospital stay. The usual case is discharged the following day after having voided, whereas those with suction drains remain for an extra day of observation. Three cases have had a mucosal perforation including cases 4, 6, and 20, none of which leaked postoperatively. There has been 1 complication, that of a distal ureteral necrosis in case 5 which necessitated open revision with a Boari flap. In this case, the ureter was held on prolonged traction with a Babcock clamp, which is no longer used. None of the cases have experienced postoperative voiding dysfunction.

## 4. DISCUSSION

A few technical aspects merit greater commentary, especially where there may be differences with other authors. To begin, though exposure of the ureter is fastest from the bladder up to the pelvic brim, it may be helpful for the first few cases to mobilize the ureter from the pelvic brim caudally until one is familiar with the anatomical orientation of the juxtavesical ureter. Cystoscopically placed ureteral catheters are not necessary though they were used in the first few cases to document that the ureter was not obstructed by an errant detrusor suture. The direction of the detrusorotomy should be straight up; a medial orientation will lead to kinking of the ureter whereas a lateral orientation makes for tedious dissection of the submucosal tunnel. The inverted Y-type detrusorotomy is used sparingly so as to limit the chances of mucosal injury. Instead, the detrusor tunnel edges are reapproximated with sutures further away from the ureterovesical junction, so as to limit ureteral obstruction by compression. If there is tension with the closure, a limited inverted Y-type dissection is performed.

Though all other authors describe the use of a single traction suture, this author believes that the use of 2 suspension stitches provides superior exposure of the mucosa as dissection progresses. In addition, the method of traction suture placement deserves greater attention. Most authors describe percutaneous passage of a Keith needle into the abdomen, whereas this author passes an intracorporeal suture extracorporeally with the use of a fascial closure device. This approach permits one to better judge the exit site of the stitch based on optimal exposure and orientation. The opposite and more commonly described approach commits the surgeon to an exit site before one has a chance to test the effect on bladder exposure. The direction of tunnel dissection is ergonomically best from the neohiatus downwards towards the ureterovesical junction. Unfortunately, this can lead to nuisance bleeding obscuring the exposure of the remaining mucosa. Ideally, one would want to dissect from the ureterovesical junction upwards towards the neohiatus that way the bleeding does not obscure vision, which is impossible with rigid instruments. Perhaps this is an area where the superior dexterity of the robot may be of benefit.

Considering the multiple options for the surgical management of VUR currently available, the indications for a laparoscopic extravesical approach are debated. The families electing to choose this option are concerned with the success rate of injectables and the mounting evidence that the product is not durable over the long term. These families want a successful procedure so as to avoid multiple postoperative VCUG’s or to minimize the risk of another pyelonephritis in those who have experienced recurrent pyelonephritis. The advantages of reduced pain and convalescence are less in the infant population such that the procedure is offered mainly to school age children. Cosmetic considerations become more important in the postpubertal population. As a result of this selection process, the case load is smaller relative to the overall cohort of surgically managed VUR, which does impact on operative time.

Having chosen a laparoscopic approach, other considerations include whether to use a transvesical approach or an extraperitoneal approach. Though the extravesical approach is ideally suited to an extraperitoneal exposure, this author feels that the extra surgical time involved in creating the space is not warranted. When one considers that the bowel is not mobilized and that the peritoneal window used for transperitoneal exposure is so small, it is difficult to imagine significant adhesions occurring in such a context. I have been impressed in the cases converted open at how small the peritoneal window was; in fact the bowel did not enter the wound. Likely for these reasons, there are no published reports on extraperitoneal ureteral reimplantation, though extraperitoneal pelvic laparoscopy has been reported for various procedures [8].

The transvesical approach with pneumobladder was first described by Okamura et al. with the technique of endoscopic trigonoplasty [9]. This procedure has been abandoned both by the original authors and others [10–13]. The idea of a pneumobladder was advanced with the initial attempts at endoscopic Cohen procedure [12, 14]. This approach has gained popularity [15–17], likely due to concerns over voiding dysfunction with bilateral extravesical surgery. The largest series to date was recently reported by Canon et al. [18] with acceptable outcomes, though the success rate was less than open surgery. It remains to be seen if the morbidity of laparoscopic unilateral transvesical surgery is greater than the laparoscopic extravesical approach, similar to the open experience. Despite a large case load and experience, Canon et al. still needed a bladder catheter for at least 36 hours, likely due to the multiple bladder perforations. In addition, with proper patient selection, the extravesical approach can be used bilaterally without voiding dysfunction. Our favorable experience with laparoscopic bilateral extravesical ureteral reimplantation is corroborated by that of Lakshmanan and Fung [6] and that of McAchran and Palmer [19] with the open extravesical approach.

Laparoscopic extravesical ureteral reimplantation was popularized by Lakshmanan and Fung [6] with excellent outcomes in their series of 47 patients and 71 ureters. They reported a 100% resolution rate of VUR, though operative times were not documented. Unfortunately, they also experienced 3 cases of distal ureteral necrosis and emphasized that the Babcock clamp should not be used for control of the ureter. Based on this author’s personal experience as well, I would strongly concur. Since then, Shu et al. [20] have published excellent outcomes in a postpubertal cohort of 6 female patients. They comment on how the laparoscopic approach to the pelvis is relatively easier than open pelvic surgery in adolescents, an opinion shared by this author. The excellent outcomes with extravesical reimplantation have been further corroborated in a more challenging set of patients including duplicated ureters [21], dismembered ureteral tailoring [22], and psoas hitch as an adjunct [23].

Nevertheless, in most series, the occasional problem of mucosal perforation and its attendant prolonged catheter drainage persists in comparison to open extravesical surgery. It remains to be seen if the ergonomic advantage of robotic assistance will be helpful in this regard. Improvements in instrumentation such as a hook electrocautery which is shielded posteriorly and thus does not cause mucosal perforation by thermal injury would be of tremendous benefit. Furthermore, prospective experimental study of the facility of mucosal exposure at different insufflation pressures and different bladder filling volumes deserves greater attention.

## 5. CONCLUSIONS

Laparoscopic extravesical ureteral reimplantation is another option in the surgical management of vesicoureteric reflux. It offers a greater success rate and durability compared to injection therapy, while offering cosmetic and convalescence advantages over open surgery in the older child. The learning curve of the procedure is reasonable and facilitated by an analysis based on 4 components, namely, access, ureter exposure, tunnel dissection, and tunnel closure. The component of tunnel dissection is the only one which could benefit from further improvement, likely accomplished with refinements in instruments or greater study of the variables which contribute to mucosal perforation.
